# Do fish get wasted? Assessing the influence of effluents on parasitic infection of wild fish

**DOI:** 10.7717/peerj.5956

**Published:** 2018-11-13

**Authors:** Christyn Bailey, Aurélie Rubin, Nicole Strepparava, Helmut Segner, Jean-François Rubin, Thomas Wahli

**Affiliations:** 1 Department of Infectious Diseases and Pathobiology, Vetsuisse Faculty, Centre for Fish and Wildlife Health, University of Bern, Bern, Switzerland; 2 University of Applied Sciences, Hepia, Geneva, Switzerland; 3 Maison de la Rivière, Tolochenaz, Switzerland

**Keywords:** Wild fish, Aquatic pollution, Wastewater, Multiple stressors, Host-parasite interactions, PKD, *Tetracapsuloides bryosalmonae*

## Abstract

Many ecosystems are influenced simultaneously by multiple stressors. One important environmental stressor is aquatic pollution via wastewater treatment plant (WWTP) effluents. WWTP effluents may contribute to eutrophication or contain anthropogenic contaminants that directly and/or indirectly influence aquatic wildlife. Both eutrophication and exposure to anthropogenic contaminants may affect the dynamics of fish-parasite systems. With this in mind, we studied the impact of WWTP effluents on infection of brown trout by the parasite *Tetracapsuloides bryosalmonae*, the causative agent of proliferative kidney disease (PKD). PKD is associated with the long-term decline of wild brown trout (*Salmo trutta*) populations in Switzerland. We investigated PKD infection of brown trout at two adjacent sites (≈400 m apart) of a Swiss river. The sites are similar in terms of ecology except that one site receives WWTP effluents. We evaluated the hypothesis that fish inhabiting the effluent site will show greater susceptibility to PKD in terms of prevalence and disease outcome. We assessed susceptibility by (i) infection prevalence, (ii) parasite intensity, (iii) host health in terms of pathology, and (iv) estimated apparent survival rate. At different time points during the study, significant differences between sites concerning all measured parameters were found, thus providing evidence of the influence of effluents on parasitic infection of fish in our study system. However, from these findings we cannot determine if the effluent has a direct influence on the fish host via altering its ability to manage the parasite, or indirectly on the parasite or the invertebrate host via increasing bryozoa (the invertebrate host) reproduction. On a final note, the WWTP adhered to all national guidelines and the effluent only resulted in a minor water quality reduction assessed via standardized methods in this study. Thus, we provide evidence that even a subtle decrease in water quality, resulting in small-scale pollution can have consequences for wildlife.

## Introduction

Proliferative kidney disease (PKD) has been strongly associated with the long-term decline of wild brown trout (*Salmo trutta*) populations in Switzerland (Burkhardt-Holm, 2002; Wahli et al., 2002, 2007). PKD is caused by the myxozoan parasite *Tetracapsuloides bryosalmonae*. *T. bryosalmonae* has a two-host life cycle, comprising of salmonid fish (the vertebrate host) and freshwater bryozoa (the invertebrate host). Infective *T. bryosalmonae* spores are released from the bryozoans into the water. On encounter of a suitable fish host, spores infect the fish through the gills and/or skin and are then transported by the circulatory system to the target organs, primarily the kidney (Clifton-Hadley, Bucke & Richards, 1987; Hedrick, Monge & De Kinkelin, 1992). In the kidney, *T. bryosalmonae* penetrates the interstitial tissue, multiplies and differentiates from extrasporogonic to sporogenic stages (Longshaw et al., 2002). During PKD infection, a huge renal swelling and mortality may occur in severely diseased fish (Kent & Hedrick, 1985; Hedrick, MacConnell & De Kinkelin, 1993).

In Switzerland, elevated water temperatures have been suggested to exacerbate PKD infections and drive disease related mortality of wild brown trout (Wahli et al., 2002, 2007). Moreover, several lab and field studies have demonstrated the influence of temperature on host-parasite dynamics in the salmonid—*T. bryosalmonae* system (Bettge et al., 2009a, 2009b; Bailey et al., 2017, 2018; Strepparava et al., 2017). However, given the strong interference of human activities with the diversity and functioning of freshwater ecosystems (Søndergaard & Jeppesen, 2007; Vörösmarty et al., 2010; Dodds, Perkin & Gerken, 2013), PKD dynamics may not only be driven by temperature change, but by multiple stressors. In fact, a number of laboratory and field studies have already demonstrated the influence of multiple stressors upon aquatic wildlife (Johnson et al., 2007; Rohr et al., 2008; Acevedo-Whitehouse & Duffus, 2009; Buck et al., 2012; Segner, Schmitt-Jansen & Sabater, 2014) and, more specifically, for diseases of freshwater fish (Schisler, Bergersen & Walker, 2000; Jacobson et al., 2003; Peeler & Feist, 2011; Johnson & Sumpter, 2014). The cumulative impact of multiple stressors may ensue in nonlinear effects and ecological surprises (Segner, Schmitt-Jansen & Sabater, 2014).

One important environmental stressor in freshwater ecosystems is pollution. Both eutrophication and exposure to anthropogenic contaminants have been shown to affect the manifestation and dynamics of infectious diseases of various aquatic species (Johnson et al., 2007; Rohr et al., 2008; Peeler & Feist, 2011) and are able to influence fish-parasite systems (Poulin, 1992; Blanar et al., 2009; Vidal-Martínez et al., 2010). Gross aquatic pollution has been reduced in recent decades through the construction of wastewater treatment plants (WWTPs). Although depending on the quality of the WWTPs, a mix of micropollutants, microorganisms, or nutrients can make their way into the waterbody and decrease water quality (Daughton & Ternes, 1999; Heberer, 2002; Costanzo, Murby & Bates, 2005; Stackelberg et al., 2007; Glassmeyer et al., 2008). This is particularly pronounced when the dilution factor of the wastewater in the receiving freshwater system is low, as is often the case in small streams or during the dry season.

Aquatic pollution may contribute to infectious disease processes in following ways: (1) directly influencing the resistance of the host, through adverse effects on multiple physiological functions, including the immune system, (2) enhancing the replication rate of pathogens, (3) indirectly influencing the abundance and distribution of pathogens hosts and vectors and/or (4) influencing the transmission of infectious stages. For instance, micropollutants including pharmaceuticals such as diclofenac, hormonally active compounds or chemicals activating the aryl hydrocarbon receptor possess the potential to compromise the immunocompetence of fish, and thereby increase their susceptibility to pathogens (Arkoosh et al., 2010; Casanova-Nakayama et al., 2011; Arkoosh et al., 2015; Rehberger et al., 2017). Or, sewage-derived organic enrichment of sediments, resulting in bottom-up effects via increasing populations of invertebrate hosts, leading to greater parasite species richness and subsequently increased presence of parasitic infection in fish can occur (Marcogliese & Cone, 2001; Krueger et al., 2006; McKenzie & Townsend, 2007). Either way, directly and/or indirectly the presence of aquatic pollution and its potential influence on host-parasite interactions is a massive concern for wildlife and one that clearly requires much more attention.

Considering, both the evidence for the impact of PKD on wild salmonids and the influence pollution may have upon aquatic diseases, thus far only one study has attempted to explore the influence of a decrease in water quality on the salmonid—*T. bryosalmonae* host-parasite system. El-Matbouli & Hoffmann (2002) studied the effect of WWTP effluents on the prevalence of PKD infection in farmed rainbow trout (*Oncorhynchus mykiss*) and wild brown trout. They observed that infection prevalence decreased in both farmed and wild fish populations after the effluents were removed. While their study provided some initial indications that PKD may be influenced by pollution it was limited by the overall low prevalence of PKD in wild fish (El-Matbouli & Hoffmann, 2002). Taking this into account, and considering that over the last decade, greater knowledge has been generated concerning the relationship between fish disease and pollution it is undoubtedly time to readdress the PKD-pollution question and broaden our horizons relating to this potential interaction. Such a study can complement earlier findings and elucidate insight into the combined effects of a chemical and natural stressor and its effects upon wildlife.

With this in mind, an ideal study site in Switzerland is provided at the Boiron de Morges. Here two stations in close proximity (∼400 m), the Amont step and the Aval step in the river Boiron exist. These stations are almost identical in terms of ecology and both PKD positive sites, inhabited by bryozoans, brown trout, and parasites and experience virtually matching water temperatures throughout the year. Furthermore, river structure is the same. However, the caveat is that the decrease in biological water quality from the Amont to the Aval step triggered by WWTP effluents may be too subtle to have a clear-cut impact on PKD.

The goal of this study was to explore the implications of altered water quality for parasitic infection of wild fish. More specifically, we tested the hypothesis that brown trout inhabiting the stretch downstream to the WWTP effluent (Aval step) will show greater susceptibility to PKD infection than trout at the upstream site. We investigated susceptibility by (i) infection prevalence, (ii) parasite intensity, and (iii) host health: in terms of disease driven pathology. We predicted that PKD infection prevalence, parasite intensity and the impact on host health in terms of pathology would be increased in fish at the Aval step. In addition, we evaluated (iv) estimated apparent survival rate and mortality of fish at each site. We expected that at the Aval step there would be a decreased apparent survival rate as an outcome of the increase in PKD susceptibility, leading to greater mortality in comparison to the Amont step. For these purposes, the field campaign consisted of monthly samplings at both sites from June to October 2017. Infection prevalence and parasite intensity were measured via concentration of parasite DNA copies in the fish kidney. Pathological changes in the kidneys of infected fish were examined by histopathology. Furthermore, we used the capture-mark-recapture method to estimate apparent fish survival and mortality at each site.

## Materials and Methods

### Study sites

**Figure 1:**
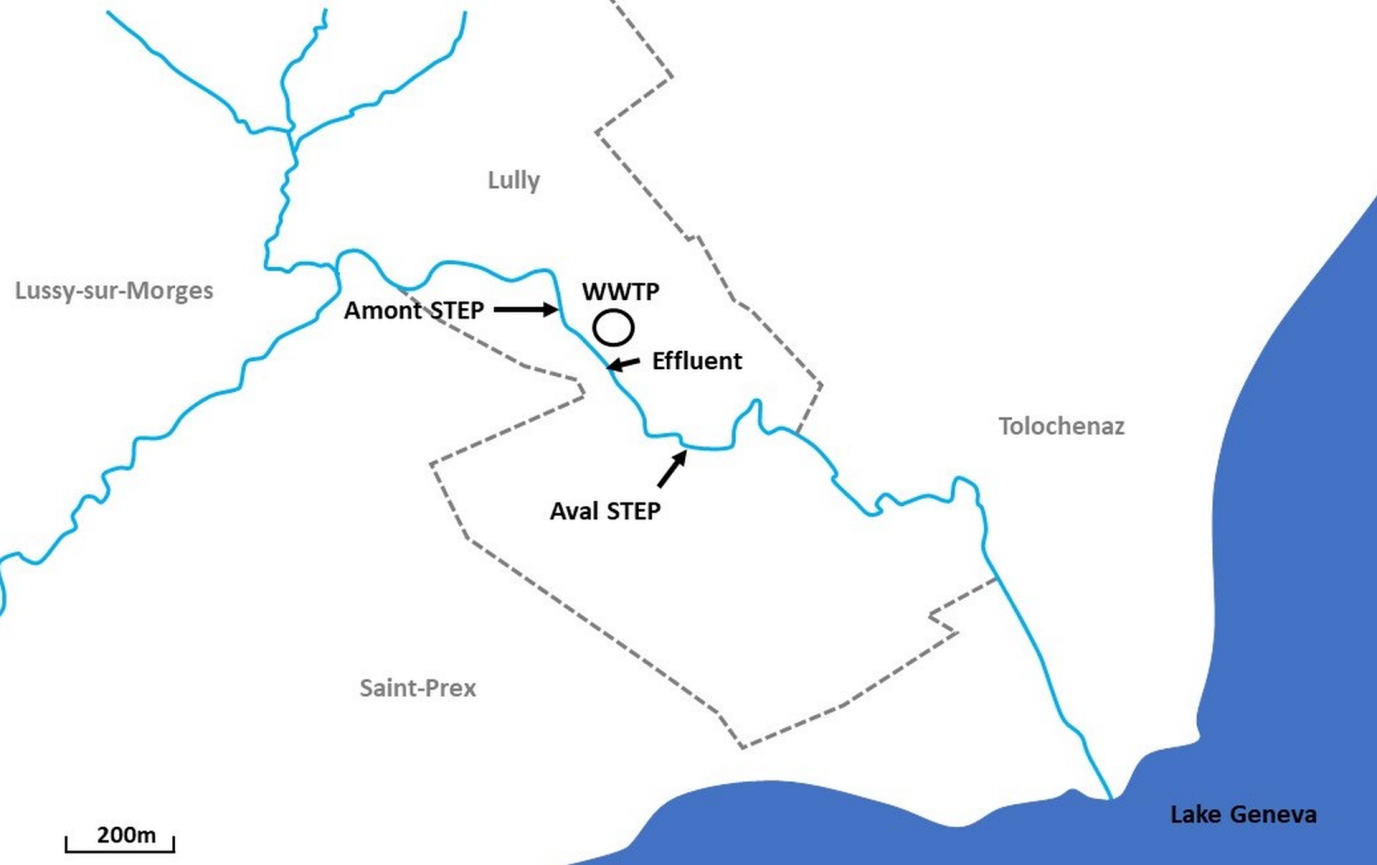
Location of sampling sites along the Boiron in the canton of Vaud, Switzerland. Two sites, a short distance apart (∼400 m) were sampled: the Amont step (46°29′56.171″N 6°28′01.204″E) and the Aval step (46°29′47.961″N 6°28′13.301″E). Also included is the location of the wastewater treatment plant (WWTP) (46°29′52.513″N 6°28′05.500″E) and the effluent (just below the highway).

Samplings were performed at the Boiron de Morges in the canton of Vaud, Switzerland. The Boiron (14 km long) is a stream that flows into Lake Geneva. Two sites, at a short distance apart (∼400 m) were sampled: the Amont step (46°29′56.171″N 6°28′01.204″E) and the Aval step (46°29′47.961″N 6°28′13.301″E) (Fig. 1). No tributary reaches the stream between these two sites, except the arrival of the WWTP effluent (46°29′52.513″N 6°28′05.500″E) upstream from the Aval step (also Fig. 1).

While there is no barrier preventing migration between the Amont and Aval, based on almost 20 years of field investigations performed at the Boiron; the majority of young-of-the-year (YOY) brown trout do not migrate between the sites until they reach juvenile or adult stages (J-F. Rubin, 2018, personal communication). In our study, we only investigated YOY fish. YOY fish are defined as age 0 fish, that is, those fish born within the year of sampling. Moreover, based on electrofishing and a fish tagging investigation described in detail within this study, only three out of 69 YOY fish tagged at the Amont step were detected downstream at the Aval step, whereas only one out of 99 YOY fish tagged at the Aval step was found upstream at the Amont step. Therefore, we exclude migration between the sites as a confounding variable. This means we consider only fish sampled at the Aval step to be influenced by the WWTP.

### WWTP effluent

The Aval step receives effluent from the WWTP of Lully–Lussy, which treats sewage from 1,412 inhabitants, with no industries or hospitals in the area, the majority of the waste water treated at the WWTP originates from households (Direction générale de l’environnement (DGE), 2017). The WWTP uses a combined fluidized bed reactor and activated sludge system. The WWTP is equipped for nitrification (removal of nitrogen) and a reed bed sludge treatment system.

During period of low-water level, the “Q347” of the river Boiron (defined as the flow rate which, averaged over 10 years, is reached or exceeded on an average of 347 days per year and which is not substantially affected by damming, withdrawal, or supply of water, that is, 95% of the time) at the level of the WWTP is 42 l/s. The dilution ratio between wastewater and river water is 1:14 (1 volume of wastewater per 14 volume of river water). This indicates the effluent represents 7% (1/14 = 7%) of the water flow.

At the WWTP outlet, the concentration of the 5 day biochemical oxygen demand, which represents the biodegradable organic matter, reaches a concentration of 4.0 mg O_2_/l. The output for the chemical oxygen demand that quantifies the oxidizable materials has a value of 32 mg O_2_/l. The organic carbon intensity reaches a concentration of 8.0 mg C/l. Finally, a concentration of 1.1 mg N/NH4 is measured at the WWTP outlet (Direction générale de l’environnement (DGE), 2017). All these values correctly adhere to the national standards enacted by the Federal Office for the Environment (FOEN). In this manner, nationally as well as internationally the effluent is not deemed an illegal or in no circumstances major source of pollution (Direction générale de l’environnement (DGE), 2017). However, a mixture of micropollutants, microorganisms, excessive nutrients and/or metals in low concentrations may remain untreated or poorly treated within the WWTP and make their way into the waterbody and thus decrease water quality.

### Water quality assessment

To determine the difference in water quality, a standardized method according to the guidelines of FOEN was used. This technique uses the IBCH index (Indice biologiquesuisse - The standardized biological index adapted to Switzerland) which indicates the ecological status of the site taking into account water quality, habitat morphology, and hydrology via, the evaluation of macrozoobenthos (Federal Office for the Environment (FOEN), 2011). Depending on the sensitivity to water quality of the observed taxon, a score between 0 (very poor ecological quality) and 20 (excellent ecological status) is defined for each investigated site. Samplings were performed in March 2015. The General Directorate of the Environment from the canton of Vaud (DGE) collected and determined the material from the Amont step station while we performed the sampling and analysis of the Aval step station. Macrozoobenthos material was sampled according to field study approvals from the DGE.

### Water temperature

Water temperature is an essential mechanism modulating PKD-induced clinical signs and mortality (Bettge et al., 2009a, 2009b; Bailey et al., 2017, 2018; Strepparava et al., 2017). Importantly, if water temperature is very much identical at both sites, then any difference in PKD infection dynamics is not driven by water temperature but by another factor. To confirm that the water temperature was comparable at both sampling sites throughout the sampling year, it was examined with temperature loggers (HOBO® Water Temp Pro v2 Data Logger, Onset, Cape Cod, MA, USA) recording data every 15 min over a period for the year. In addition, we also compared the amount of days with water temperatures ≥15 °C, as this critical temperature threshold is linked with elevations in PKD related mortality (Ferguson, 1981; Clifton-Hadley, Richards & Bucke, 1986).

### Fish sampling

No restocking is done at either site, therefore all fish sampled in the study are wild. Monthly samplings from June to October 2017, were performed at each site. At each sampling 25 YOY brown trout per site were captured by electrofishing over a section of 100 m long (*N* = 125 experiment total per site). YOY fish sampled here would have hatched from eggs spawned in March, being around 3-months-old at the first sampling (Rubin, 2018, personal communication). Therefore, these fish would have had a fully competent immune system (Magnadóttir et al., 2005). After capture, fish were euthanized using 3-aminobenzoic acid ethyl ester (MS 222®; Argent Chemical Laboratories, Redmond, WA, USA). Total length and total weight were recorded. The kidney was carefully removed and cut in two halves longitudinally: one part was weighed (kidney weight, KW) and stored in one ml RNAlater (Qiagen, Basel, Switzerland) for downstream DNA extraction, while the other half was put in formalin to be prepared for histology. Fish and ICBH samplings were carried out relating to ethical approvals (Service of Consumption and Veterinary business of the canton of Vaud, permission number VD3253).

### Fish tagging

In July at both sites, additional YOY fish were captured and tagged with a passive integrated transponder-tag before release. The tags allowed us to track fish movements using a portable antenna and investigate apparent survival rates and fish mortality at each site. A total of 69 YOY fish from the Amont step and 99 YOY fish from the Aval step were captured and tagged. After capture, fish were anesthetized (MS 222) and a small cut with a scalpel was performed above the pelvic fins. The tag was injected in the abdominal cavity and its unique number was obtained with a reader.

In October, tagged fish were recaptured to determine the apparent survival rate, which is calculated as the number of tagged recaptured fish divided by the total amount of tagged fish. At the same sampling before returning the recaptured fish to the sampling sites, we used the portable antenna to detect the remaining tags. All detected tags found on the river floor were considered as dead fish and thus the percent mortality was calculated from these samples. Individual fish not recaptured or detected were deemed to have emigrated. It could be assumed that these individuals may have dispersed and died, although this is impossible to confirm. Thus, for the sake of the study, these fish were defined as emigrated. In addition, through monitoring movement of tagged fish allowed us to investigate if migration was occurring between the sampling sites.

### Determination of infection prevalence and parasite intensity

Proliferative kidney disease infection prevalence and parasite intensity were assessed by qPCR. Approximately 25 mg of tissue was used for DNA extraction. DNA extractions were carried out using the Blood and Tissue DNA extraction kit (QIAGEN, Basel, Switzerland) following manufacturer’s guidelines. Then DNA was eluted with 100 µl EB buffer, provided in the kit and stored at −20 °C, until qPCR was carried out. qPCR was performed with primers and probe according to Strepparava et al. (2017) for this model, using an Applied Biosystem analyser (Applied Biosystems, Rotkreuz, Switzerland). The qPCR was carried out in a final volume of 20 µl containing 1× TaqMan universal Master Mix (Applied Biosystems, Switzerland), 0.5 μM of each primer (PKDtaqf1: 5′-GCGAGATTTGTTGCATTTAAAAAG-3′ and PKDtaqr1: 5′-GCACATGCAGTGTCCAATCG-3′), 0.2 μM of the probe PKD (5′-CAAAATTGTGGAACCGTCCGACTACGA-3′) labelled with FAM-TAMRA, 1× of IC DNA (TaqMan Univ. MMix w Exog IntPostC; Applied Biosystems, Switzerland), and two μl of template DNA. Standard curves were generated for each qPCR cycle using plasmids containing the amplified fragment. For all plate 5 logs of plasmid dilution standards were amplified (from 10^6^–10^2^ gene copies number). To concur reliability of the qPCR, the coefficient of the standard regression had to be in the range −3.6 to −3.0 as per the manufacturer’s instructions (Applied Biosystems, Switzerland), the coefficient of variation of quantification within each standard and sample in duplicate had not to surpass 25% and the non-target control (water) had to show no amplification (Mackay, 2004; Joly & Bruneau, 2006; Yun et al., 2006; Hellemans et al., 2007). If reliability criteria were not met, the qPCR was repeated. Percent prevalence was calculated per each time point, while parasite intensity (DNA copy number) was taken for each individual and standardized to KW.

### Histological assessment

Following routine processing and paraffin embedding, kidney sections of 3–5 µm thickness were prepared on SuperFrost® Plus positively charged glass slides. The slides were stained with hematoxylin and eosin for histological examination. Histopathological alterations throughout the kidney sections were assessed, these included, tissue proliferation, infection degree (presence and distribution of parasites), and presence of fibrous tissue. All parameters were scored individually 0–6. The degree of tissue proliferation in terms of proliferation/pathological alterations was scored as: 0 (none), 1 (scattered), 2 (mild), 3 (mild to moderate), 4 (moderate), 5 (moderate to severe), or 6 (severe), modified from (Schmidt-Posthaus et al., 2012). Concerning infection degree: 0 indicated no presence of parasites, whereby a 6 specified a very high number of parasites per view. Relating to the presence of fibrous tissue: fibrous tissue is an indicator of the tissue regeneration process which is stimulated to recover/regenerate organ structure and function. Its presence has been observed in salmonids recovering from PKD infection (Schmidt-Posthaus et al., 2012, 2017). Therefore, a strong score in this category from one sample site in comparison to the other sample site may indicate a fish in recovery stage. Presence of fibrous tissue was thus also scored from 0–6 using the same index as tissue proliferation (Schmidt-Posthaus et al., 2012). This scoring system was used for statistical investigation between the two sites. One slide was evaluated per fish.

To further evaluate the variance in infection dynamics between the sites we examined the relationship between infection variables host health and parasite intensity. In this manner, we used increasing tissue proliferation as a proxy for decreasing host health during infection, as increasing proliferation in the kidney during PKD infection results from an enhanced inflammatory response and associated nephritis, corresponding with host immunopathology and even mortality.

### Statistical analysis

The statistical differences between sites were tested for significant differences using a *t*-test. Correlations between host health and parasite intensity were assessed by calculating the Pearson product—moment correlation coefficient (*r*). All tests were performed using SigmaPlot 12.0 (Systat Software, San Jose, CA, USA) and graphically presented with GraphPad Prism 5 (GraphPad Software, Inc., San Diego, CA, USA) Excel 2010 (Microsoft, Redmond, WA, USA) or SigmaPlot 12.0. Significance was set at *P* ≤ 0.05.

## Results

### Water quality assessment

As an integrative parameter to assess the ecological status at the Amont and Aval sites, we used the IBCH index, which is based on the evaluation of macrozoobenthos. Using this methodology, the Amont step reached a score of 14, and the Aval step a score of 12—on a scale ranging from 0 (very poor ecological quality) to 20 (excellent ecological status). Based on this data, the ecological status at both sites appears to be moderate to good, as indicated from the IBCH scores, and, importantly, there is only a subtle difference between the two sites.

### Water temperature assessment

**Figure 2:**
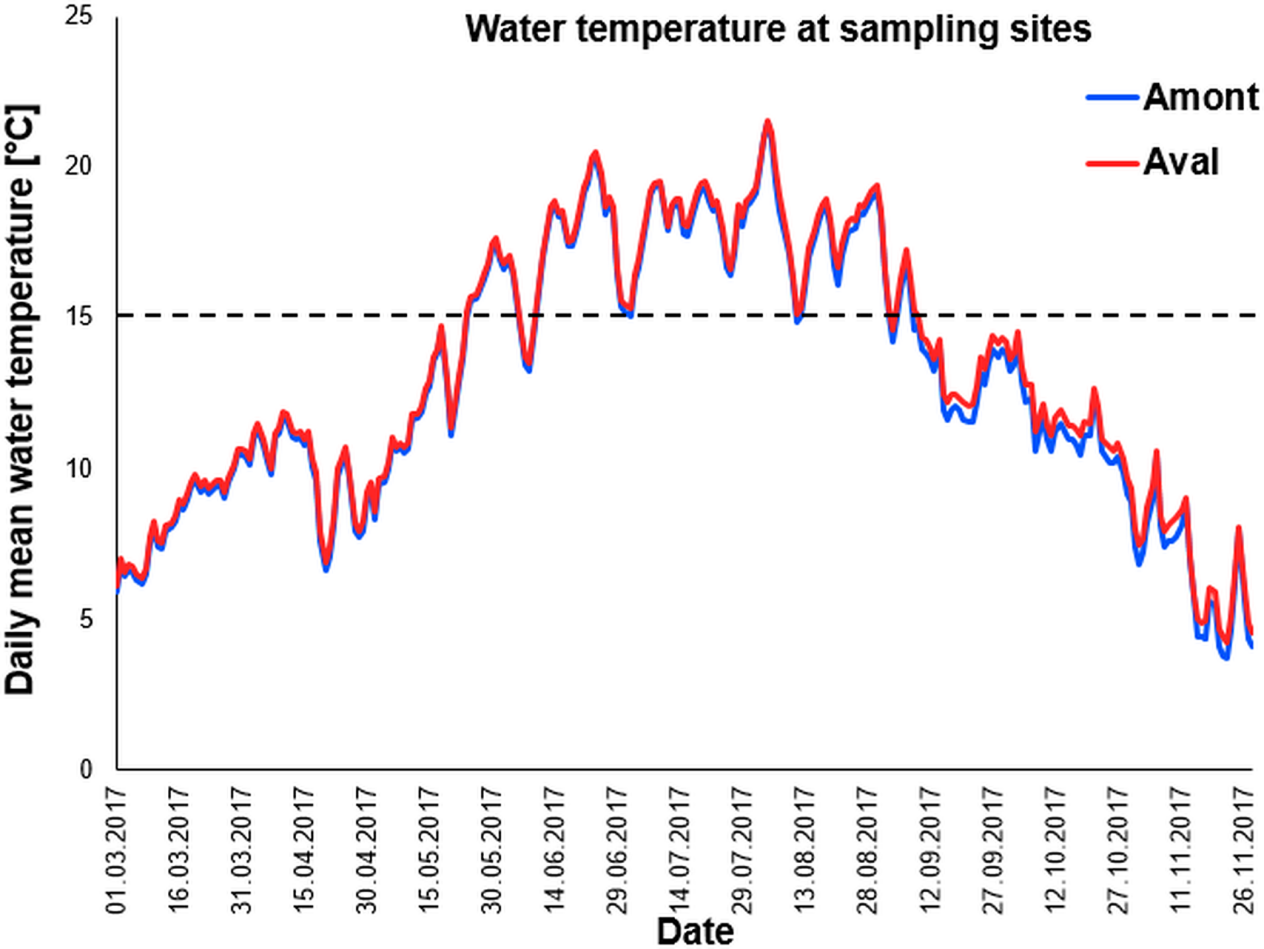
Daily mean temperature of sampling sites. Water temperature curves measured throughout the year at the sampling sites the Amont step (blue line) and the Aval step (red line). Black dotted line indicates 15 °C, the critical water temperature for proliferative kidney disease-related clinical signs and mortality in trout.

We used data loggers to monitor water temperature at the two study sites. As temperature is a key driver of PKD infection and disease severity it was critical to our study that water temperature was nearly identical at the sites. Importantly water temperatures recorded throughout the sampling campaign and beyond; March until November 2017 followed an almost identical pattern at the two study sites (Fig. 2). Furthermore, when comparing the total number of days with daily mean water temperature ≥15 °C, there was no significant difference between the sites (Table 1).

**Table 1:** Total number of days with daily mean water temperature ≥15 °C at each sampling at Amont and Aval step.

Monthly sampling	Amont	Aval
June	16	17
July	36	37
August	60	61
September	88	90
October	99	104

**Note:**

15 °C is the critical water temperature for proliferative kidney disease-related clinical signs and mortality in trout.

### Infection prevalence and parasite intensity

**Figure 3:**
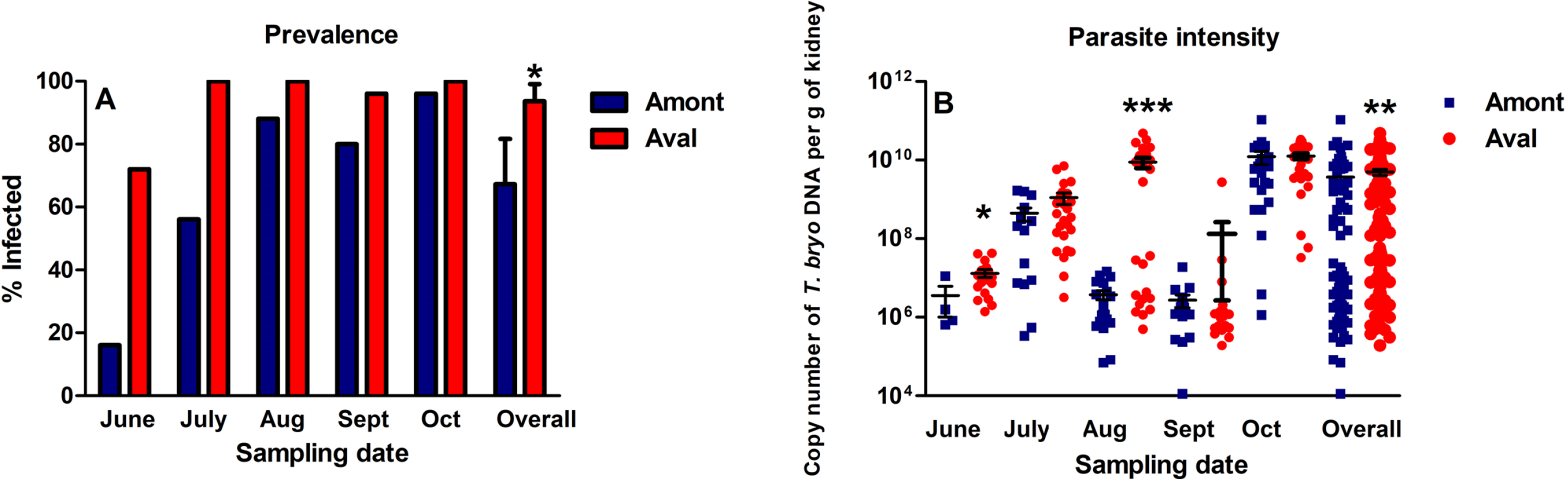
Proliferative kidney disease dynamics observed in infected fish sampled at the Amont and Aval step. (A) Infection prevalence and (B) Parasite intensity (black lines indicate mean ± SE) at sampling point of the Amont (blue bars (A) or blue squares (B)) and the Aval (red bars (A) or red circles (B)) sites. Parasite intensity was determined using copy numbers of parasite DNA as a proxy. Asterisks indicate levels of significance (*t*-Test), *P < 0.05, **P < 0.01, and ***P < 0.001. *N* = 25 per site, per monthly sampling per site.

Over the course of the experiment PKD infection prevalence at the Amont step ranged from 16% to 96%, whereas at the Aval step prevalence ranged from 72% to 100% (Fig. 3A). Overall PKD infection prevalence, consisting of data from all monthly samplings was significantly greater at the Aval step, relative to the Amont step. The parasite intensity of fish sampled at the Aval step was also significantly increased in June and August and overall in a comparison of all infected samples relative to the Amont step (Fig. 3B).

### Fish weight

**Figure 4:**
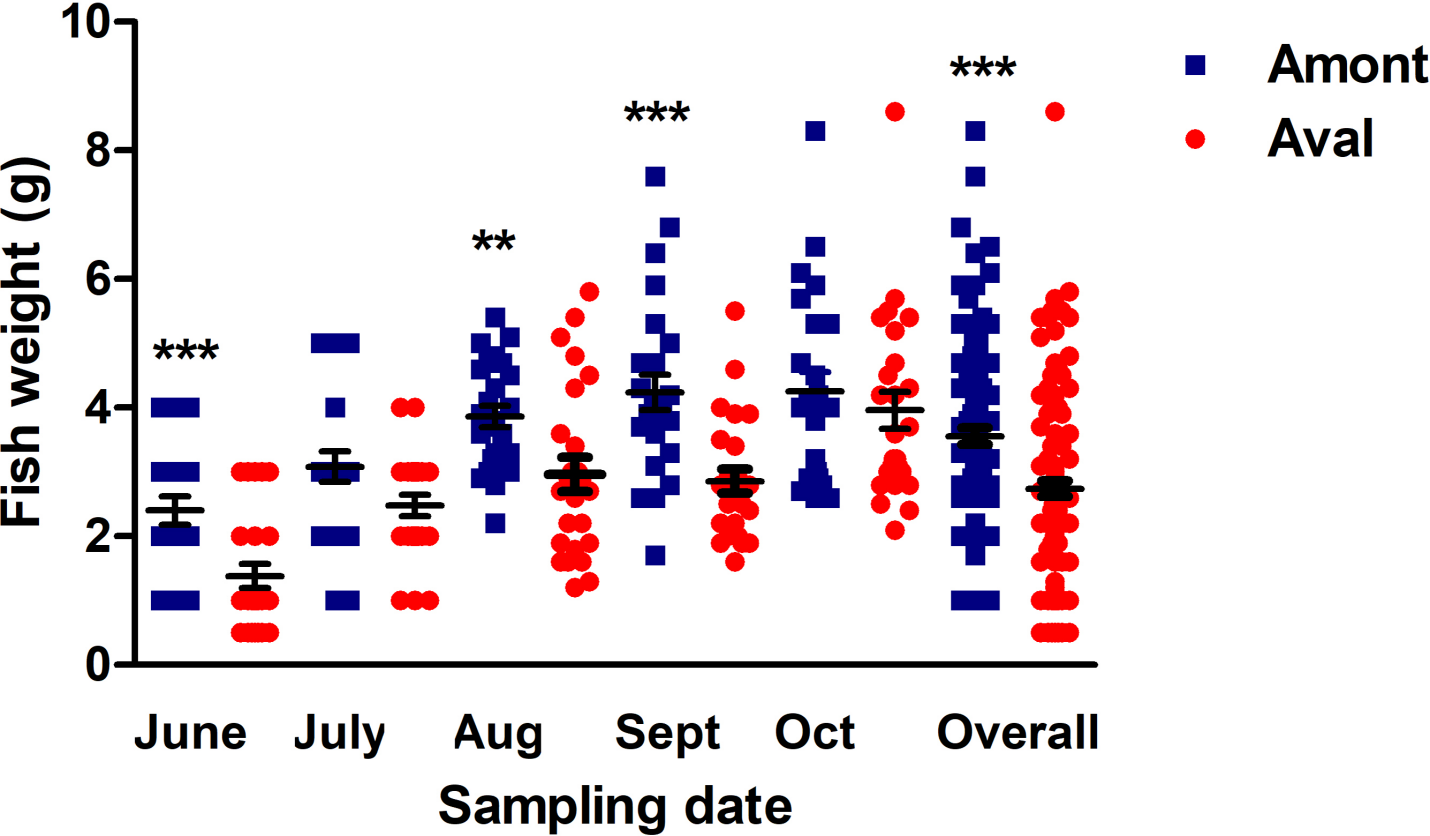
Fish body weight at the Amont and Aval. Amont (blue squares) and Aval (red circles) (black lines indicate mean ± SE) at different sampling points. Asterisks indicate levels of significance (*t*-Test), *P < 0.05, **P < 0.01, and ***P < 0.001. *N* = 25 per site, per monthly sampling per site.

Fish body weight was compared between the sites to investigate if there was an impact of the effluent on the growth of YOY fish at the Amont or Aval step. The mean weight of fish sampled at the Amont step was greater at every sampling. More specifically, at the Amont step there were significant increases in fish weight at the June, August, and September samplings and overall in a comparison of all samples relative to the Aval step (Fig. 4).

### Histological assessment

Here is an overall description and summary of the significant histology results. For a complete outline of the histology scores refer to Table 2. Owing to size of the YOY fish at the June sampling no histology samples were taken. From August parasite induced microscopic alterations of the kidney associated with PKD infection of salmonids were observed in fish at both sites. These included proliferation of the interstitial tissue, granulomatous inflammation, multiple areas of necrosis and haemorrhage, necrotising vasculitis, formation of thrombi, and fibrous tissue.

**Table 2:** Histopathological changes of the kidney section observed in infected fish sampled at the Amont and Aval step concerning infection degree, tissue proliferation, and fibrous tissue.

Site	Monthly sampling	Infection degree	Tissue proliferation	% of fish with tissue proliferation	Fibrous tissue	% of fish with fibrous tissue
Amont	July	1.2 ± 0.36	1.33 ± 0.18	33	None	None
	August	1.89 ± 0.24	1.81 ± 0.21	78	1.87 ± 0.35	32
	September	2.35 ± 0.38	2.00 ± 0.28	78	1.91 ± 0.38	69
	October	3.05 ± 0.32	3.00 ± 0.22	82	2.46 ± 0.33	65
	Overall	2.12 ± 0.32	2.03 ± 0.22	68	2.08 ± 0.35	55
Aval	July	1.83 ± 0.24	1.77 ± 0.23	50	None	None
	August	3.33 ± 0.15*	3.04 ± 0.12***	100	2.1 ± 0.31	60
	September	3.16 ± 0.19***	3.26 ± 0.21**	96	2.26 ± 0.34	62
	October	3.4 ± 0.40	3.12 ± 0.25	77	2.35 ± 0.24	91
	Overall	2.93 ± 0.98***	2.79 ± 0.20*	81	2.23 ± 0.29	71

**Notes:**

No infected fish at either site had presence of fibrous tissue in July sampling. Asterisks indicate levels of significance (*t*-Test).

* *P* < 0.05.

** *P* < 0.01.

*** *P* < 0.001.

**Figure 5:**
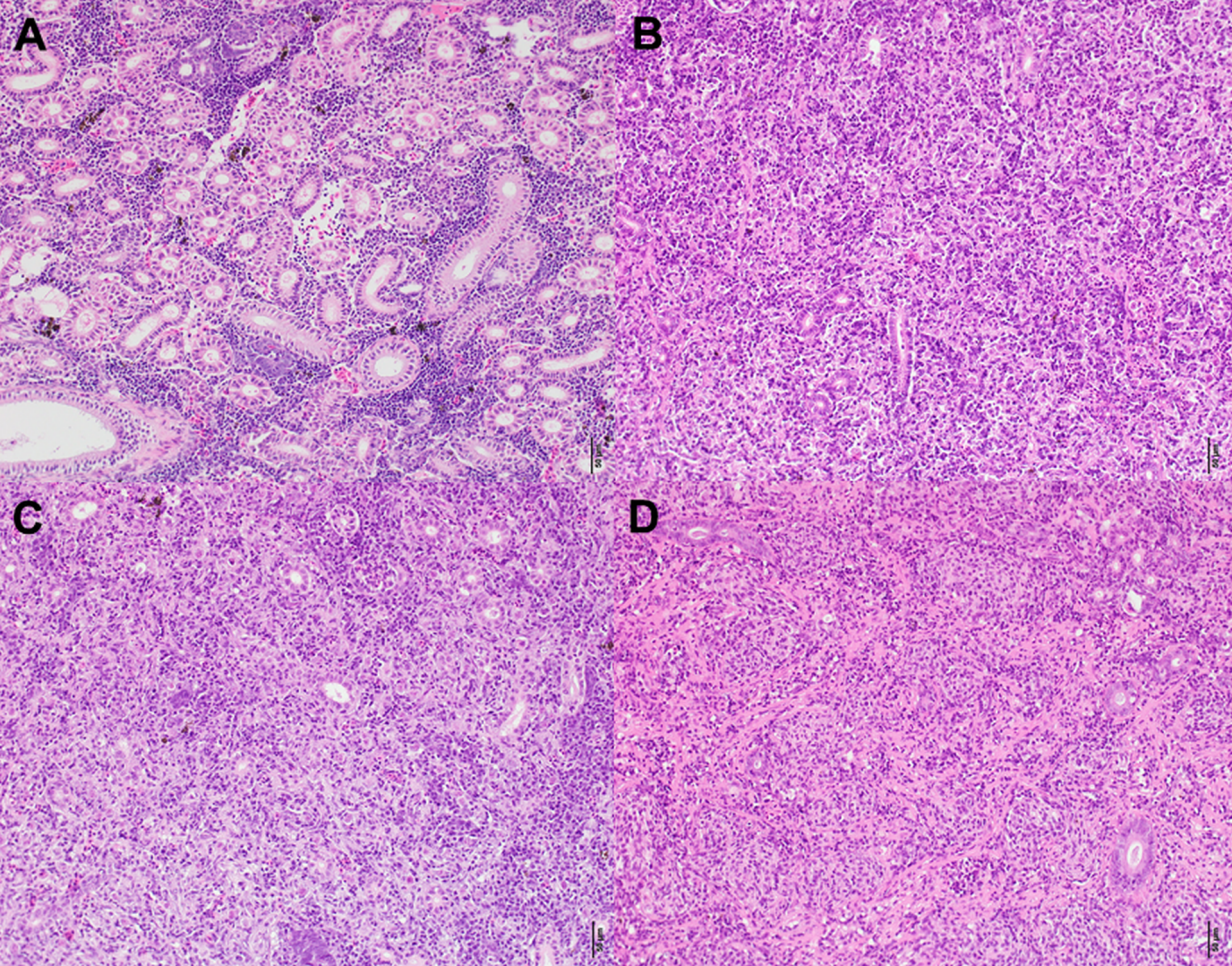
Histologicalimages of the posterior kidney. (A) Demonstrates a zero in all categories according to the histological assessment and (B–D) the highest scores in each category. (A) Is from a fish sampled at the Amont in June. (B) Is from a fish sampled at the Aval in August showing a score of six for infection degree (presence and distribution of parasites). (C) Is also from a fish sampled at the Aval in August showing a score of five (moderate to severe) for tissue proliferation. (D) Is from a fish sampled at the Amont in September showing a score of five for fibrous tissue. Scale bar = 50 μm. All pictures are taken from slides stained with H&E.

**Figure 6:**
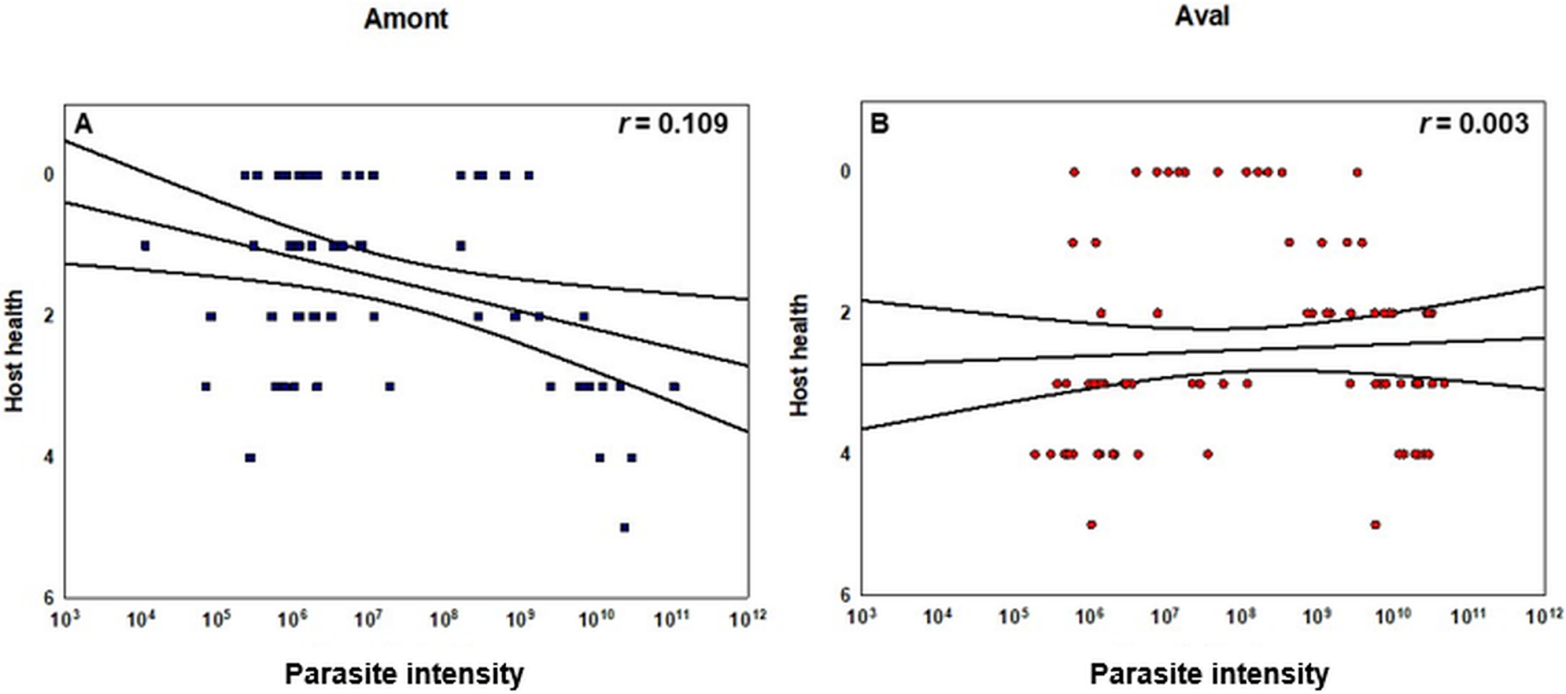
Correlation of copy number of *T. bryosalmonae* DNA per gram of kidney tissue and host health at each sampling site. (A) Amont step (blue squares) and (B) Aval step (red circles). Pearson correlation coefficients *r* as well as confidence intervals are given (black lines). Tissue proliferation score determined via histology and used as a proxy for host health. *Y-*axis is inverted to indicate that an increase in tissue proliferation is associated with a decrease in host health. There were no significant correlations between the variables at either sampling site. For the Amont step *N* = 63 and for the Aval step *N* = 82.

Concerning the statistical differences observed when examining alterations in the severity of infection; in terms of renal proliferation and degenerative alterations evaluated via tissue proliferation scores, there were significant increases in these scores from fish sampled at the Aval step relative to fish sampled at the Amont step in August and September and in overall in a comparison of all infected samples. In addition, there was a significant increase in the degree of infection, observed in terms of parasite presence per slide at the Aval step relative to the Amont step in August and September and overall in a comparison of all infected samples. By assessing the amount of parasites observed histologically it allows us to correlate the increase in parasite intensity seen by qPCR when comparing the Aval and the Amont providing further evidence of the differences in parasite intensity when comparing the two sites. Concerning the presence of fibrous tissue no significant differences were found between the two sites, at any of the samplings. Images illustrating a zero score and the highest observed in the case of each category of the histological assessment are shown (Fig. 5). The relationship between parasite intensity and host health in terms of tissue proliferation was plotted and compared statistically for each site, although, there was no significant relationship between the variables at either site (Fig. 6).

### Apparent survival, mortality, emigration, and migration

**Figure 7:**
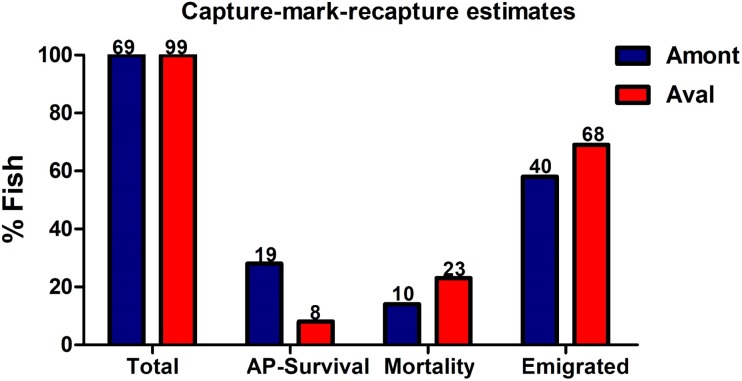
Estimates generated from the capture-mark-recapture method using PIT tags of fish at the Amont and Aval step. Estimations include apparent (AP) survival rate, mortality and possible emigration at Amont step (blue) and Aval step (red). While could be assumed that emigrated fish may have died post dispersal, though this is impossible to confirm, thus, for the sake of the study, these fish were defined as emigrated. *N* is indicated above the respective bar.

In July 2017, 69 YOY fish at the Amont step and 99 YOY from the Aval step were captured and tagged before being returned to the waterbody (Fig. 7). In October 2017 during the recapture, 19-tagged fish were recaptured at the Amont step and eight at the Aval step. The estimated apparent survival rate reached 28% at Amont step relative to 8.0% at Aval step, thus indicating a greater apparent survival rate at the Amont step. In addition, mortality calculated from detected tags found lying on the river floor was greater at the Aval step in comparison to the Amont step (23–14%). While the percent of fish deemed to have dispersed (emigration) was also greater at the Aval step (68%) in comparison to the Amont step (40%).

## Discussion

Here, we assessed the ecological implications of WWTP effluents on parasitic infection of wild fish. We evaluated the hypothesis that brown trout inhabiting the lower water quality site (Aval step) will show greater susceptibility to PKD. We assessed susceptibility by (i) infection prevalence, (ii) parasite intensity, (iii) host health in terms of pathology, and (iv) estimated apparent survival rate. At different time points during the study, significant differences between sites concerning all measured parameters were found, thus providing evidence of the influence of effluents on parasitic infection of fish in our study system. In addition, fish at the Amont step had increased body weight at all the time points investigated. While fish weight could be influenced by a swollen kidney. However, given the fish in the Amont step were heavier but had less alterations, this underpins a better growth at his particular site.

Taken together our results indicate that the fish inhabiting the Aval step had greater susceptibility to PKD infection. The main goal here was not to find major differences in terms of disease severity or mass mortality events, but to provide information on what impacts a subtle difference in ecological quality may have for this host-parasite system. Assessment of environmental pollution often focuses on medium to large-scale impacts and the influence of small-scale effluent discharges on the natural environments can be overlooked. The novelty of the present study is that it investigates these small-scale impacts and their role in PKD from the perspective of the fish host.

In our study system, the impact of effluents may have influenced PKD through a direct and/or in-direct process. Relating to the brown trout—*T. bryosalmonae* host-parasite system these processes may occur under the following synopsis: (1) through the fish host via altering its ability to manage the parasite, (2) through the parasite via effects on virulence, transmission, or reproduction, or (3) through the invertebrate host via increasing bryozoa (the invertebrate host) reproduction. Concerning the above synopses, it could be suggested that this is due to: (1) through the fish host via its ability to manage the parasite being lowered. It is widely documented environmental pollution can interfere with host physiology and/or immune processes (Colborn & Clement, 1992; Kavlock et al., 1996). In this context, a lower water quality induced modulation of the host physiology or immune response may not be dramatic *per se*, but it may lead to immunosuppression and as a result increased susceptibility to infection (Kimber & Dearman, 2002). This outcome would offer support to what we have seen in the present study: that the subtle difference in water quality caused via effluents does not lead to an extreme outcome or mass mortality event but increases of susceptibility possibly via an impact on immunocompetence.

On the other hand, it does not mean we can simply speculate that if we had seen a greater deterioration in water quality it would necessarily correlate to a greater negative outcome for the fish host. An example of this is a report evaluating the effects of environmentally relevant polybrominated biphenyl ethers (PBDEs) on Chinook salmon (*O. tshawytscha*) and the impacts associated with susceptibility to infectious bacterial diseases (*Vibrio anguillarum*) (Arkoosh et al., 2010). In this study, Arkoosh et al (2010) found that fish fed the 1× PBDE diet showed significantly greater disease-related mortality when challenged with a pathogen than control fish. In contrast, the Chinook salmon fed the 10× PBDE diet were less susceptible to disease than the fish fed the control diet. The 1× PBDE diet was proposed to have a concentration reflecting contaminant levels found in gut contents of wild Chinook salmon (Arkoosh et al., 2010). The authors did not know the reason for this result; it could be conceivable that it is due the impact of PBDEs directly on the bacteria, or indirectly through an impact on the host in terms of composition and metabolic activity of the gut microbiota, which acts as the niche for the bacteria. Either way, the study outcome demonstrates that the net impact of multiple stressors may ensue in ecological surprises. In a further example of an unexpected outcome a controlled lab multiple stressor experiment incorporating *T*. *bryosalmonae*, Burki et al. exposed fish to an E2 (estrogen 17β-estradiol) concentration and *T. bryosalmonae* challenge. Remarkably, fish exposed only to the parasite had greater parasite intensity and increased mortality relative to the fish exposed to both E2 and *T. bryosalmonae* (Burki et al., 2013). Therefore, it could be suggested that in the Burki study, E2 may have either benefited the host or hindered the parasite, in contrast to our results in which decreased water quality had a negative effect on the host (indirect through a pro-parasitic impact or directly upon host homeostatic processes). Though in our study system the WWTP effluents will contain a temporally varying combination of compounds, in comparison to the aforementioned lab study that used a pre-determined concentration of E2, hence a more drastic impact upon the host might be expected.

To stay with the role of pathogen, it might be suggested that the increase of PKD susceptibility at the Aval step maybe due to synopsis: (2) through an influence on the parasite through enhanced virulence. To avoid any conflictions, we define virulence as the host’s parasite-induced fitness loss referring to the degree of pathology caused by the pathogen. In this manner, we discuss that the differences we observed in pathology between the Amont and Aval step were due to a difference in the parasites impact on host health. Micropollutants found in effluents may include nutrients (especially carbon, nitrogen, and oxygen), and various metals; which have been shown to act as mediators of the metabolism in pathogenic bacteria and important regulators of their virulence mechanisms (Somerville & Proctor, 2009; Rohmer, Hocquet & Miller, 2011). Envisaging this, WWTP effluents may support the expression of virulence factors of pathogens (Alizon et al., 2009). For instance, it has been shown that increases in nutrient levels can moderate putative virulence factors of the salmonid pathogen *Flavobacterium columnare,* resulting in elevated expression of tissue degrading enzymes chondroitinase and collagenase, which play a role in skin lesions, fin erosion, and gill necrosis (Penttinen et al., 2016). Thus, the differences in pathology in our system may have been due to increased parasitic virulence. While *T. bryosalmonae* is able to persistently exploit bryozoan hosts by undergoing host-condition dependent development (cycling between virulent overt and avirulent covert infections), little is known in terms of identifying virulence factors and other adaptations, despite its relevance as a disease of both aquaculture and wild fish. As PKD infection of brown trout is characterized by persistent infections selection for low virulence may occur for this opportunistic parasite (Okamura, 2016). Therefore, suggesting this synopsis may be the most speculative. However, as the environment outside the host is unpredictable, such opportunistic pathogens as *T. bryosalmonae* have to adapt rapidly to the changing conditions, for example, water temperature (Guijarro et al., 2015), or even WWTP effluents, which may alter the parasite characteristics and also its impact upon the host. In the case of PKD, and its association between an increase in disease severity and mortality with elevated water temperatures, clearly *T. bryosalmonae* as an opportunistic pathogen is influenced by changes in the environment. However, if micropollutants or nutrients in the water may have a similar influence on the parasite clearly requires greater elucidation.

Finally, we consider that the differences in susceptibility we have seen are due to an influence of effluents on: (3) through the invertebrate host, via increasing bryozoan reproduction. A bryozoa survey along the Boiron de Morges performed in 2014 by H. Hartikainen and J-F. Rubin (2018, personal communication) described a greater abundance of bryozoa at the Aval step in comparison the Amont step. Furthermore, Hartikainen et al. (2009) utilizing a combination of both lab and field studies demonstrated that greater nutrient levels promote both parasite and bryozoa growth resulting in a greater number of spores available for infection of fish. Therefore, the increased nutrient intake due to the WWTP effluent at the Aval step may have promoted growth of the bryozoans and led to higher parasite densities for fish infection, similar to the results of Hartikainen et al. (2009).

Concerning other myxozoan diseases, there are several examples in which the relationship between eutrophic-like nutrients enriched environments and the intermediate hosts (in PKD the bryozoa) which suggest that the parasite biomass is also influenced by bottom-up effects (Marcogliese & Cone, 2001; Krueger et al., 2006; McKenzie & Townsend, 2007). Granting this, while all these studies clearly identify a link between an increase of intermediate host populations and greater presence of parasites via pollution enrichment and diseased fish, they do not investigate the impact on fish health, making it difficult to understand how infection severity of the fish host may be moderated by nutrients in these myxozoan diseases.

## Conclusion

While from our results we can indicate that there is a negative impact for the fish, we cannot clearly disentangle the exact synopsis (1, 2, or 3) or combination thereof in which the host-pathogen interaction is modulated. This would require dedicated lab experiments encompassing each player in the PKD life cycle to be conclusive. Moreover, our field study only investigated two sites and only through including an increased number of replicates would have allowed us to confirm with greater certainty if pollution was the true causation for the differences reported here. This is a common issue that impacts environmental studies when attempting to link a particular factor with disease without suitable replicates. However, we do provide critical baseline information for the development of future studies focusing on the impact of a subtle decrease in water quality and point to the importance of studying pollution on a small scale.

In closing, our results indicate that environmental pollution should be considered alongside temperature as a driver of PKD infection of wild populations of brown trout even if it occurs on a small-scale. In this context, while the pathogen may be the ultimate factor in diseases related mortality there are still clearly other factors that may drive mortality and to comprehensibly understand these effects we need to integrate multiple stressors studies if we are to attempt to disentangle the current and growing threats biodiversity faces.

## Supplemental Information

10.7717/peerj.5956/supp-1 Supplemental Information 1 Histology individual fish scores

## Additional Information and Declarations

### Field Study Permissions

The following information was supplied relating to field study approvals (i.e., approving body and any reference numbers): Samplings were carried out relating to ethical approvals (Service of Consumption and Veterinary business of the canton of Vaud, permission number VD3253).

### Data Availability

The following information was supplied regarding data availability: All other raw data is included within the article and in Supplementary File 1.
